# Remote Monitoring of Sustained Low-Efficiency Dialysis (SLED) Machines in Intensive Care Unit

**DOI:** 10.1016/j.xkme.2022.100452

**Published:** 2022-03-18

**Authors:** Gerard A. Zasuwa, Jerry Yee, Karla D. Passalacqua, Stanley Frinak

**Affiliations:** Division of Nephrology and Hypertension, Henry Ford Health System, Detroit, Michigan

**Keywords:** Intensive care dialysis alarms, medical alarms, remote monitoring

## Abstract

The Henry Ford Health System provides patients with a safe, improved system of continuous kidney replacement therapy using a proprietary, 24-hour sustained low-efficiency dialysis (SLED). The SLED system utilizes regional citrate anticoagulation (RCA) in conventional hemodialysis machines that have been configured to provide slow dialytic therapy. Within our hospital complex, SLED-RCA systems are deployed in intensive care units distributed over 4 floors in 2 buildings. This widespread footprint represents a spatial challenge for hemodialysis technicians. Fifteen SLED-RCA machines may be running at one time, and each deployed unit may signal an alarm for multiple reasons. Previously, audible alarms prompted intensive care unit nurses to identify the alarming machine and manually notify technicians by telephone. Technicians would then travel to resolve the alarm. To improve the process of addressing SLED-RCA machine alarms, we developed a remote alert alarm system that wirelessly notifies hemodialysis technicians of specific machine alarms. A quality improvement analysis of nearly 1,000 SLED-RCA alarms over a 1-week period revealed that the average time for alarm correction with a remote alert alarm system was approximately 5 minutes. Reducing alarm resolution time may free technicians and nurses for other critical duties.

Intensive care unit (ICU) nurses who monitor patients must respond to medical device alarm signals that “indicate unsatisfactory physiological patient states, unsatisfactory functional states of medical electrical equipment or medical electrical systems or to warn the operator of hazards to the patient or operator due to the medical electrical equipment or medical electrical system.”^1^ An analysis of medical alarms at the Johns Hopkins Hospital documented a total of more than 59,000 alarms over a 12-day period, or 350 alarms per patient per day.^2^ Too many alarms can result in health care provider alarm fatigue. Cropp et al^3^ examined whether physicians, nurses, and respiratory therapists could differentiate audible alarm signals in an ICU. Their analysis revealed that of 33 different sounds, only 50% of the critical alarm signals and 40% of noncritical alarm signals were correctly identified. In 1999, one study identified 40 distinct alarms signaled by medical devices for a single ICU bed.^4^ The addition of hemodialysis machine alarms to this high acuity setting further adds to alarm complexity and poses a challenge to overall patient safety. Furthermore, hemodialysis machine alarms are loud, often exceeding 80 dB, and the noise can lead to patient sleep deprivation,^5^, ^6^, ^7^ a problem inherent to acute care settings. The added noise also represents an additional stressor for health care providers.^8^^,^^9^ Thus, initiatives that reduce the alarm burden in the ICU are greatly needed.

Up to 7% of ICU patients require continuous kidney replacement therapy, compounding the potential sensory overload in the ICU from dialysis machines.^10^ Many ICU dialysis machine sensors are hardwired to a central nursing station, affording remote patient monitoring. However, hemodialysis technicians and nurses who control and monitor hemodialysis machines work in a mobile fashion, and dialysis personnel usually use equipment that is not hardwired to a centralized monitoring station. Given the critical and prolonged nature of continuous kidney replacement therapy, central monitoring is essential for optimally safe care delivery. We previously described the safety and utility of modified conventional hemodialysis machines running at low blood flow rates and showed that 24-hour sustained low-efficiency dialysis (SLED) is a feasible treatment approach.^11^^,^^12^ At our institution, our proprietary SLED-regional citrate anticoagulation (RCA) machines are deployed continuously, typically for 5 or more days, and the slower blood flow rate of 60 mL/min requires regional citrate anticoagulation (SLED-RCA) to prevent extracorporeal circuit clotting.

Because SLED-RCA treatments are continuous, it is impractical and inefficient to assign a dedicated staff member to monitor each machine. In particular, the COVID-19 pandemic caused an increased demand for SLED-RCA units, which put pressure on the overworked, struggling staff in an overcrowded ICU. In the absence of a centralized alarm system, correcting dialysis machine alarms is often delayed. The amount of time for dialysis machine alarm resolution in the ICU has been inconsistent and attributable to unfavorable onsite staff-to-machine ratios. Also, ICU nurses are not universally trained in hemodialysis technology and generally are not expected to resolve hemodialysis machine alarms; therefore, “simple fixes” that need minimal technical input still require the appearance of a hemodialysis technician to resolve the problem.

Our continuous kidney replacement therapy treatment protocol requires dialysis personnel to go on rounds to chart the SLED-RCA machines at 2-hour intervals, and personnel who conduct SLED-RCA therapies are not continually present in patient rooms to monitor treatments. When a SLED-RCA machine alarms due to changes in machine parameters, adverse consequences for the patient could occur; therefore, systems that shorten the time for responding to alarms are needed. To prevent iatrogenic adverse events in patients receiving dialysis, we developed and implemented a real-time sentinel remote alert alarm system to address SLED-RCA machine alarms with the aims of responding quicker to alarms and enhancing protection of critically ill patients undergoing SLED-RCA. We implemented the remote alert alarm system at our institution in 2015 and have consistently optimized the system to a point where we believe it is functioning sustainably. Thus, we performed a quality improvement analysis to characterize the remote alert alarm system. We assessed the number and types of alarms as well as the mean alarm response time and queried dialysis machine technicians about their perceptions of the system. Here, we describe our remote alert alarm system: how it is set up, how it works, and the benefits that we believe it has provided to clinical care at our institution. We hope that this quality improvement narrative will inspire other institutions to implement similar systems to optimize care of patients who require hemodialysis.

### Description of SLED-RCA

Our proprietary protocol for SLED-RCA requires 2 intravenous infusion pumps. Citrate is infused into the arterial blood line of the extracorporeal circuit just after the connection to the central venous hemodialysis catheter. Before blood is returned to the patient’s circulation, a second intravenous pump normalizes the ionized calcium via an infusion of a calcium- and magnesium-containing solution into the venous return line of the circuit. Fig 1 illustrates the various parts of SLED-RCA, which are referred to in the following description of the system and how it may signal an alarm in the clinic.

**Figure 1:**
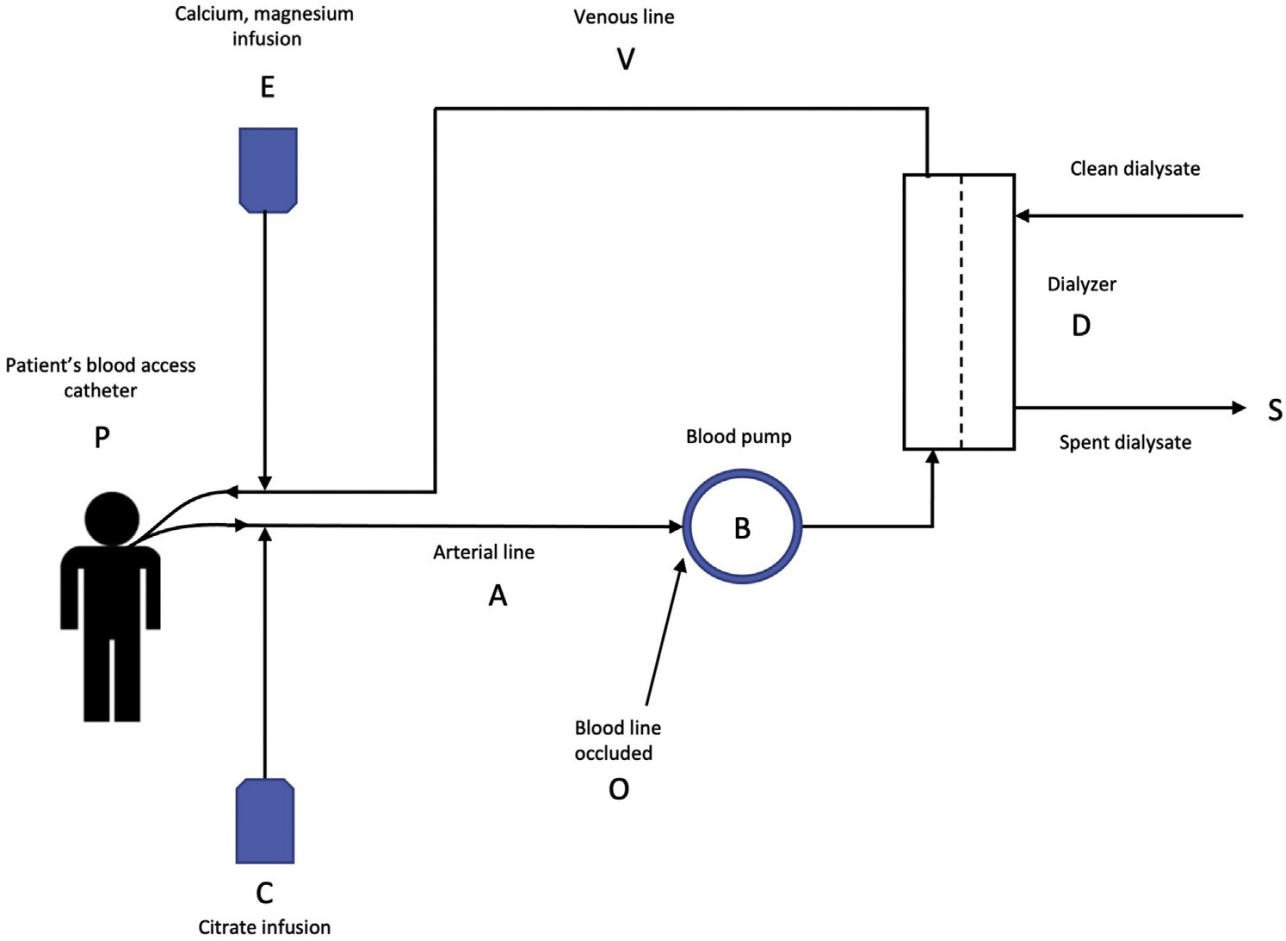
Regional citrate anticoagulation during normal operation of the dialysis machine. Abbreviations and definitions: A, Atrial line; B, blood pump; C, citrate infusion; D, dialyzer; E, calcuim magnesium infusion; O, blood line occluded; P, patient’s blood access catheter; S, spent dialysate; V, venous line.

If blood flow through the extracorporeal circuit is stopped, the blood pump (B) rollers will automatically occlude the arterial blood line. However, the first infusion pump will continue to deliver citrate (C) that directly enters the patient’s systemic circulation via the arterial branch of the hemodialysis catheter, which can be life-threatening if it leads to depressed plasma ionized calcium levels. If the SLED-RCA machine alarm system detects an error, which could include issues involving temperature, composition of the dialysate, conductivity, blood leak, and others, the machine will alarm and enter a “bypass” mode, where dialysate does not enter the dialyzer and the dialytic process is suspended until the alarm is cleared. A list of dialysis machine alarm triggers is listed in Box 1. During bypass alarms, the blood pump does not stop, and blood continues to flow in the extracorporeal circuit to prevent clotting; however, since the citrate-calcium complex is not being removed from the blood, the patient’s ionized calcium level will fall. Under these conditions, citrate (C) is infused into the arterial blood line (A), passes through the dialyzer (D) without citrate being removed, and then flows into the venous line (V) and the patient (P).

Box 1 **List of Dialysis Machine Alarms that Technicians May Receive Through the Remote Alert Alarm System**

**Table:** 

Temperature
Dialysate flow
Blood pump function
Reagent level detector
Arterial Pressure
Venous Pressure
Transmembrane Pressure (TMP)
Blood leak
Blood pressure
Conductivity
**Custom Alarms**
Small molecule clearance (Kecn)
Blood flow not equal to treatment QB
Treatment ending in 10 min

Blood from the patient (P) flows into the arterial blood line (A) where citrate (C) is infused. Plasma containing citrate-calcium complexes passes through the blood pump (B) for clearance by the dialyzer (D) as spent dialysate (S). The blood exits the dialyzer and enters the venous line (V) where calcium and magnesium (E) are infused and blood enters the systemic circulation (P). An alarm condition that stops the blood pump (B) is unsafe because the arterial line is occluded at point (O) where the pump roller contacts the arterial blood line. Under this condition, citrate is infused into the systemic circulation of the patient (P) via the arterial blood line (A). A bypass mode alarm condition that stops the flow of dialysate is unsafe because dialysis is suspended and citrate is not removed. The blood pump (B) will continue to operate as citrate is infused into the arterial blood line (A). The blood will pass through the dialyzer (D) without citrate removal, into the venous line (V), and then into the patient (P). The complexity of this system and the dire consequences of malfunction underscore the need for rapid response time.

### Description of the Remote Alert Alarm System

The SLED-RCA machines at our institution are now monitored remotely with the remote alert alarm system, which uses a secure internal WIFI and telephone system (ASCOM). We developed the remote alert alarm system alarm telemetry software system to provide critical, timely surveillance of SLED-RCA systems. The remote alert alarm system has been in use at our institution since June of 2015, and Fig 2 shows a model overview of the system. The Fresenius 2008T dialysis machine has an independent computer equipped with the Clinical Data eXchange software (CDX) system. The CDX system was initially intended to run medical information systems; however, we employ it as a Windows-based computer. The Nephrology Department systems analyst has 2 computers that monitor real-time functions to assure the system is functional every day.

**Figure 2:**
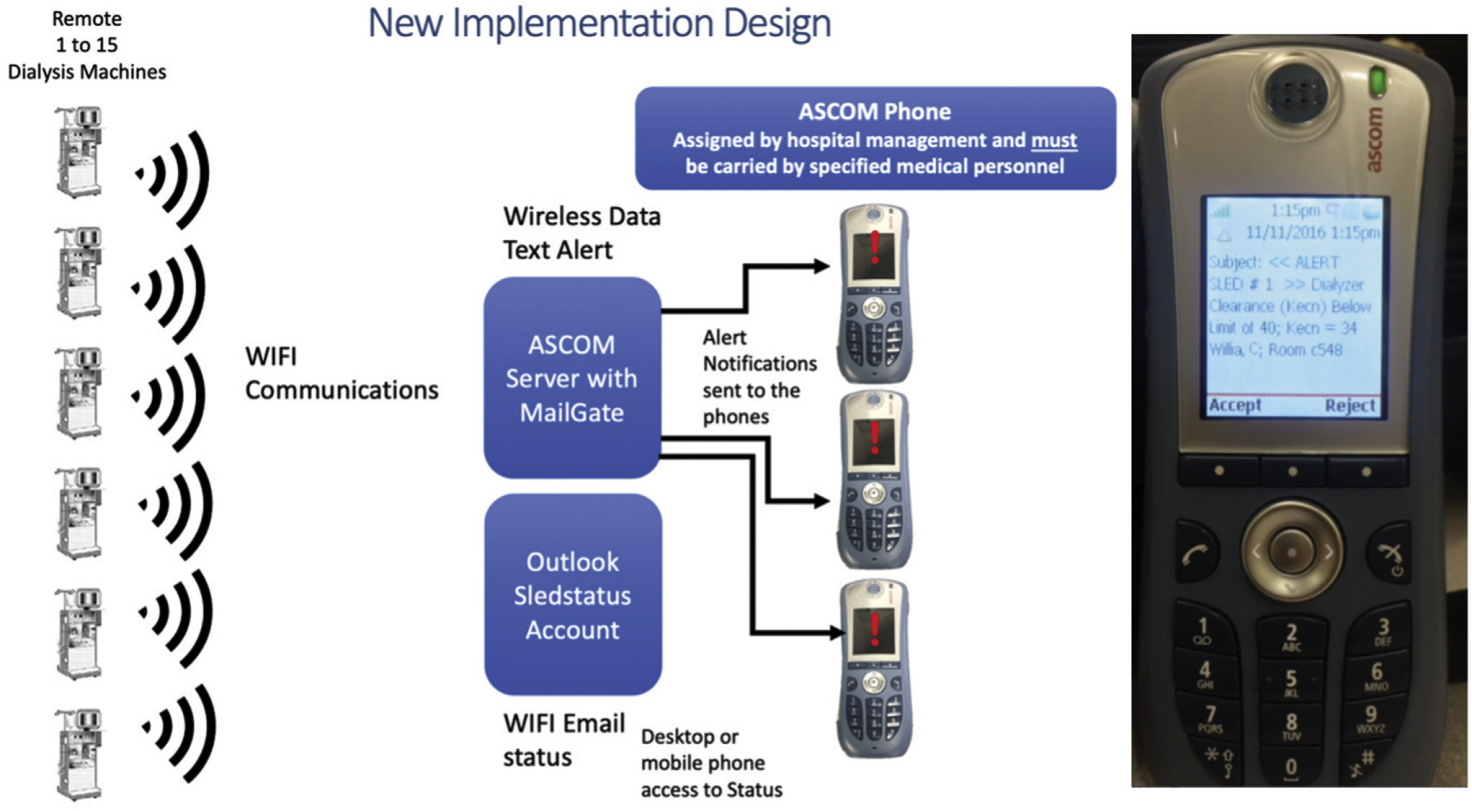
ASCOM phone integration and WIFI communications. Hemodialysis machines send wireless data text alerts to communicate with an ASCOM server with MailGate. The MailGate server forwards the alerts to ASCOM phones carried by specified medical personnel, and the text message must be accepted or rejected by personnel receiving the text message. All transactions are logged with a time stamp for documentation of the event.

To facilitate the notification of our dialysis nurses and technicians via the remote alert alarm system, we created a software instrument panel (National Instruments LabVIEW, v. 15), that displays machine and patient data from multiple hemodialysis machine/patient parameters and alerts technical staff for immediate resolution of problems by sending notifications to ASCOM phones. The ASCOM software module, MailGate, provides text alerts in specific intervals to proprietary phones until the alarm is resolved. Building on this infrastructure, we developed a system to leverage the hospital’s secure, encrypted WIFI system to deliver hemodialysis machine alarm text-alert notifications to hemodialysis technicians and nurses with ASCOM phones (Fig 2). In short, the remote alert alarm system via ASCOM provides a wireless alert system for contacting health care providers for prompt resolution of hemodialysis machine alarms. Alerts are sounded for any alarm state every minute for 5 minutes and then every 5 minutes until the alarm is resolved; thus, an alarm for the blood pump may produce 4-6 alerts or more until resolved. In addition to sending critical alerts, the real-time telemetry system also logs errors in an event log so that events can be retroactively assessed (Fig 3).

**Figure 3:**
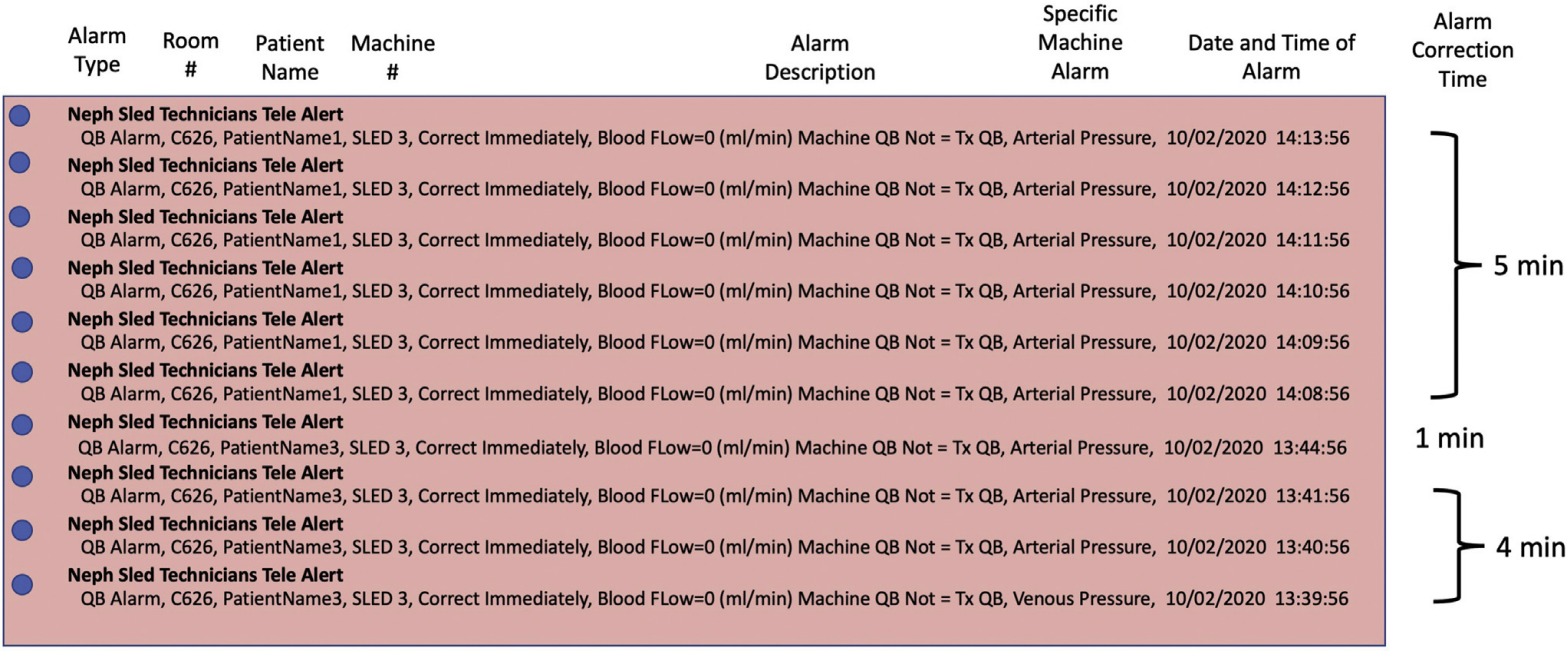
Remote alert alarm system alarm server log describes the alarm type, blood flow (QB), room number, patient name, alarm description, date, and time of alarm. The specific alarm type is the exact hemodialysis machine alarm that stopped the blood pump and created a blood flow alarm and is used to help diagnose the current alarm event. Alarm time is used to calculate the alarm correction time.

For connecting the remote alert alarm system to the dialysis machines, first-generation model Fresenius hemodialysis machines without built-in computer screens were furnished with all-in-one medical grade computers with touchscreens (Fig 4A). The external RS232 data port connection is used to collect machine data. Second-generation Fresenius hemodialysis machines have a large touch screen, and users can alternate between machine functions and computer functions readily (Fig 4B). The computer screens display real-time errors in a banner for nursing and technical staff to review and respond to.

**Figure 4:**
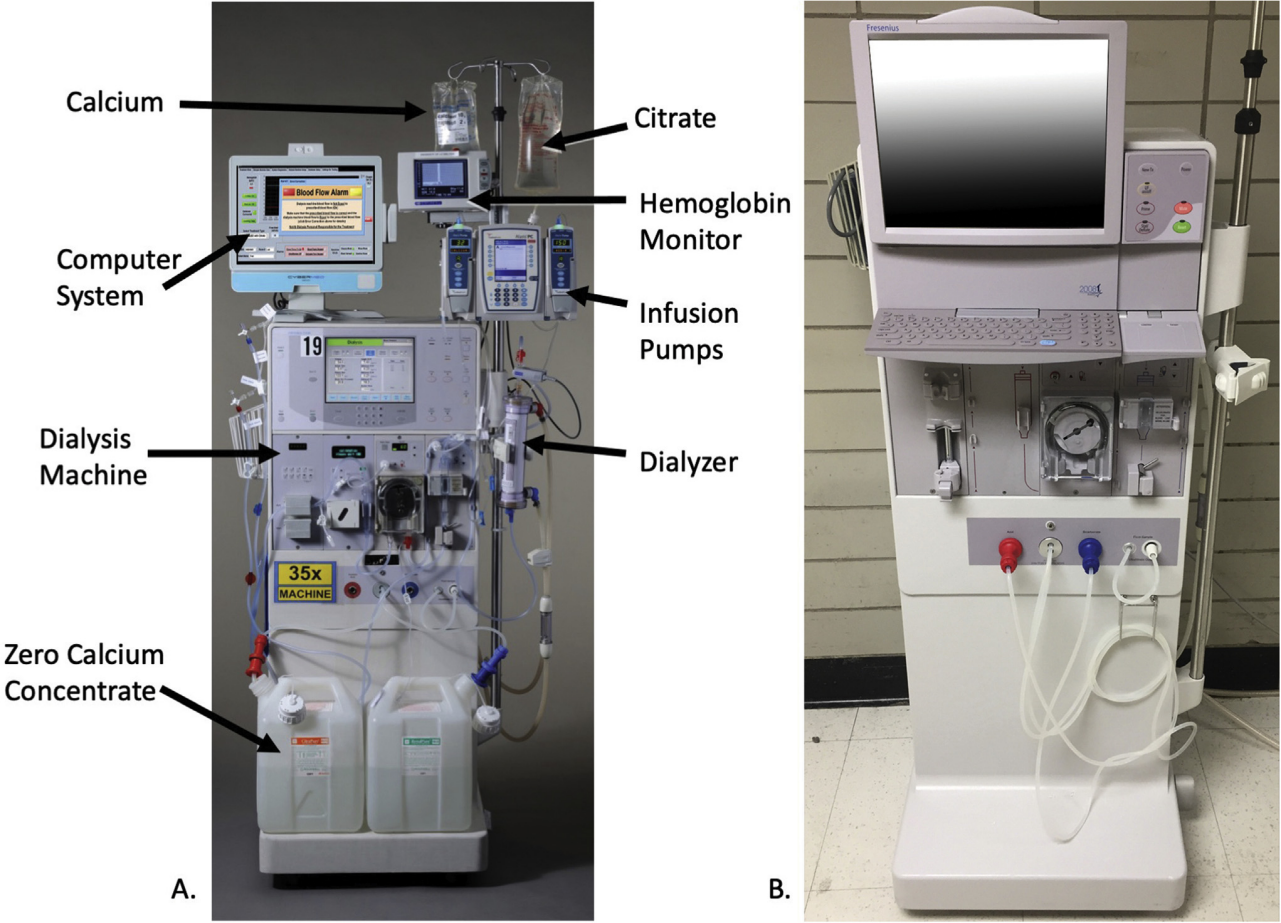
**A.** First-generation Fresenius 2008K hemodialysis machine with wireless monitoring computer and touch screen setup. **B.** Second-generation Fresenius 2008T hemodialysis machine with built-in computer and touch screen.

Before we had implemented the remote alert alarm system, if ICU nursing staff observed or heard a hemodialysis machine alarm, the nurse would first press the reset button on the machine to clear the alarm. If that did not clear the alarm, the nurse would then call for assistance from a hemodialysis technician. With the remote alert alarm system, the hemodialysis technician first receives an alert on an ASCOM phone and then calls the ICU nurse, providing guidance on how to operate the hemodialysis machine and clear the alarm. Alert messages transmitted to ASCOM phones include alarm type, patient name, room number, hemodialysis machine number, alarm description, specific machine alarm, date, and time of alarm. The specific machine alarm information facilitates the technician’s diagnosis of the alarm. For example, the alarm for the server data shown for patient 1 in Fig 3 is an arterial pressure alarm that signifies a blood flow obstruction in the arterial blood circuit. The obstruction could be in the arterial branch of the central venous catheter or within the arterial blood line itself in front of the hemodialysis machine blood pump. A second example, provided in Fig 3, shows that patient 2 has a venous pressure alarm, indicating obstruction in the venous branch of the central venous catheter or an obstruction to blood flow after the venous pressure transducer connection to the blood circuit.

To ensure that alarms are not missed by hemodialysis technicians, the remote alert alarm system transmits the same alert to the ASCOM phone every 1 minute for 5 minutes and then every 5 minutes until the alarm is cleared on the hemodialysis machine. All alarms generated for SLED-RCA machines are critical and cannot be suppressed by the remote alert alarm system. Remote alert alarm system alarms are time-stamped and saved. This storage feature permits review of the hemodialysis technician alarm response for calculating response times. The remote alert alarm system also sends messages to technicians 10 minutes before the 10-hour hemodialysis treatments are set to terminate so that supplies can be promptly brought to the machine to start the next 10-hour round of treatment time.

As further backup, our remote alert alarm system has an alarm display screen on the dialysis machine for daily checks on remote alert alarm system functionality. Because our IT infrastructure footprint is large, upgrades, server migration, and server retirement can result in interruptions of service. If our IT service provides notice of upcoming changes, we can test preemptively to avoid disruption. If the remote alert alarm system needs to be interrupted for system upgrades, dialysis personnel increase the frequency of rounding to monitor all machines.

ASCOM provides communication with an IP-messaging tool, MailGate, that interfaces to our email system. This tool delivers notification alerts directly to an ASCOM telephone or pager as soon as an email arrives. MailGate is interfaced to all email servers and clients supporting simple mail transfer protocol. Functionality, such as filtering and forwarding conditions, varies depending on available features in the existing mail server. Each telephone or pager in the ASCOM system has a unique email address and phone number. Portions of an incoming email are automatically forwarded and displayed as notifications on the unit. The staff are immediately alerted that the received notification requires attention. Overall, we deployed Microsoft Exchange for its simple mail transfer protocol and distribution group feature to email the notification alert to carriers in our group.

Patient data can be sent over the remote alert alarm system because the network is secure within the hospital. ASCOM phones can also be tied to EPIC, the hospital’s electronic medical record, so EPIC alerts can be selectively texted to the user of the phone (eg, lab results, discharges, etc). The EPIC alerts are especially useful for medical staff who are rounding in the hospital and distant from the medical device. These alerts refer to hemodialysis machines that are not functioning properly and that could possibly cause patient harm unless corrected. For example, on a typical day, up to 40 alarms on 6 hemodialysis machines might occur. As the machine population doubles or triples, the number of potential alarms escalates. Our system, which includes a medical grade computer software (developed at Henry Ford Health System) and email alerts sent via the ASCOM telephone service, eliminates patient machine errors, and reduces possible harm resulting from medical and technical errors. Another advantage of our WIFI-enabled computers running the dedicated software module is that data are transmitted to the central exchange email server at 5-minute intervals. This dedicated server creates a list of active machines and locations (Fig 5). Consequently, medical staff can identify patients and operational hemodialysis machines from any remote, in-hospital location. When a communication failure in the system does occur, ICU nurses manually call technicians to resolve SLED-RCA alarms.

**Figure 5:**
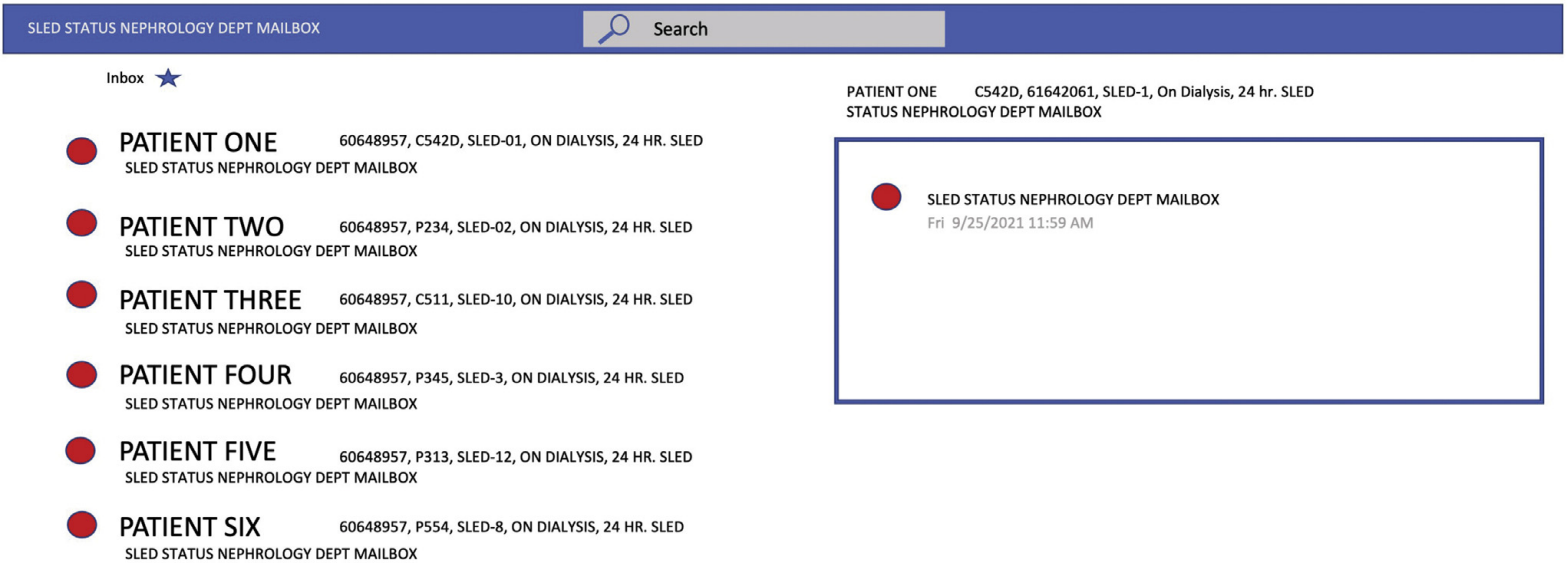
Active machine main system telemetry display.

Developing the remote alert alarm system incurred several costs. Our main investment for developing the system was in software engineering by 2 nephrology software engineers and 1 IT specialist who configured the ASCOM communication module. Overall, the entire project took 2 months of part-time effort with 2 months of testing before full implementation. The entire electronic remote alert alarm system runs unattended and requires very little maintenance. Our IT department maintains the ASCOM and Outlook architecture 24/7, while nephrology staff update and support the remote alert alarm system software.

### Remote Alert Alarm System Performance

Because the remote alert alarm system has been used and optimized since 2015, we performed a descriptive, quality improvement assessment of the system to better characterize our novel approach. We analyzed the number of alarms, the types of alarms, and the average weekly number of alarms that occurred through the remote alert alarm system in 15 SLED-RCA units between December 26, 2020 and May 12, 2021. We also calculated the mean alarm resolution time during a 1-week period when the system logged over 1,000 alarms per week (higher than the average).

The WIFI remote alert alarm system has been in use at our institution since June 2015. Infrequent failure is a hallmark of the system; however, when a communication failure in the system does occur, ICU nurses manually call technicians to resolve SLED-RCA alarms. Alert logs from 15 SLED-RCA machines during the study period included 11,896 alerts. Alerts indicated alarm situations where dialysis was suspended. The most frequent alarm involved approximately 6,000 blood pump alerts from arterial or venous pressure alarms that caused the pump to stop. The second most frequent alarm was the dialysate flow alert alarm triggered by improper conductivity or temperature, totaling 2,754 alerts. The average weekly alarm rate during the analysis period was close to 600 alerts per week.

We chose a 1-week timeframe within the study period that included nearly 1,000 SLED-RCA alarms to assess alarm resolution time and observed an average correction time of approximately 5 minutes. The baseline alarm resolution time for the previous manual phone alert system could not be calculated because technicians had not historically recorded this parameter, and the time it had taken for ICU nurses to call technicians under the previous system was unknown. However, eliminating the need for a phone call by the ICU nurse most likely saved precious time, the goal of the remote alert alarm system initiative.

### Informal Survey of Remote Alert Alarm System Users

In 2020, after the remote alert alarm system had been in use for over 5 years, we sent an anonymous, paper survey with 7 multiple choice questions to dialysis technicians to query their perceptions about the functioning of the remote alert alarm system. These questions were developed by management and nursing leaders for quality improvement purposes and were not developed to gather generalizable information.

Of the 11 technicians who were sent informal surveys regarding the remote alert alarm system, 10 responded (Table 1). All the respondents indicated that they responded quicker to SLED-RCA alarms with the alert system. While blood pump and venous pressure alarms were the most chosen as being the most useful alarms, level detector and transmembrane pressure alarms were most chosen as being the least useful alarms. Interestingly, the small molecule clearance (Kecn) alarm had 5 responses for being the most useful alarm and 5 responses for being the least useful alarm. Most respondents (6/10) indicated receiving less than 1 ASCOM alert per hour per shift and 3 said they received 1 to 3 per hour per shift. Most of the technicians (90%) agreed or strongly agreed that the ASCOM alert makes patient care safer, and all the technicians either agreed or strongly agreed that knowing which SLED machine alarming is helpful.

**Table 1:** Results of the Dialysis Technician Satisfaction Survey (N = 10)

Survey Question	Number (%) *N* = 10
Which of the following SLED alarms from ASCOM do you find most useful? (Choose all that apply) (Top 3 most chosen alarms)	
Blood pump	6
Venous pressure	6
Kecn	5
Which of the following SLED alarms from the ASCOM alert system do you find the least useful? (Choose all that apply) (Top 3 most chosen alarms)	
Level detector	5
Transmembrane pressure	5
Kecn	5
How often do you receive a SLED alarm using the ASCOM alert system per shift?	
<1 per h	6 (60%)
1 to 3 per h	3 (30%)
4 to 6 per h	1 (10%)
How would you rate the following statement, “ASCOM Alert makes patient care safer”?	
Strongly agree	3 (30%)
Agree	6 (60%)
How has the ASCOM alert affected your response time to troubleshoot major SLED alarms?	
Faster response	10 (100%)
Do you feel ICU nurse calls to resolve SLED alarms have decreased after implementation of the ASCOM alert?	
Strongly agree	2 (20%)
Agree	3 (30%)
Neither agree nor disagree	4 (40%)
Strongly disagree	1 (10%)
Knowing which SLED machine(s) is (are) alarming is helpful.	
Strongly agree	7 (70%)
Agree	3 (30%)

Abbreviations and definitions: ICU, Intensive care unit; Kecn, small molecule clearance; SLED, sustained low-efficiency dialysis.

### Impact of Remote Alert Alarm System

Here, we described a quality improvement initiative that included creating and implementing a remote alarm alert system monitoring dialysis machines to optimize the clinical response time for alarm resolution in the ICU and to maintain the highest possible level of patient safety. Remote alert alarm system wireless telemetry for dialysis machine alerts has the potential to improve patient safety, reduce technical errors, and improve the ability to correct problems rapidly. Improvement of SLED-RCA can be achieved by incorporating the essential feature of alarms, but this endeavor requires human and organizational network infrastructure to create a distributed alarm system for remote caregivers. Future improvements to be addressed include incorporating machine learning to enable intelligent decision-making around resolving alarms for transient issues.

We believe that the remote alert alarm system has optimized dialysis machine alarm response in several ways. First, nurses conducting hemodialysis in ICUs no longer need to contact a technician for each SLED-RCA alarm, saving time. Also, hemodialysis staff are directly notified by the system as to when treatments are nearing completion and when machines must be reset to continue for the next 10-hour treatment period, which may reduce treatment interruptions. Long alarm intervals can occur when a machine runs out of dialysate concentrate because resupplying the concentrate can take as long as 30 minutes. With the remote alert alarm system, the low conductivity alarm is transmitted directly to the technician, so concentrate can promptly be brought to the machine. Thus, health care providers, even when they are not in the vicinity of the units, can readily determine which patients are actively undergoing SLED-RCA and plan accordingly for maintenance and care. Also, because of the tracking and archival features of the remote alert alarm system, critical incidents and errors can be interrogated retroactively. These improvements may be real timesavers for ICU medical staff since the system encourages prompt resolution of dialysis machine alarms. Lastly, alarms, on average, are now resolved within 5 minutes, although we were not able to determine how this compares to the previous system since that system had not been tracked. Quicker alarm resolution time also reduces noise, which is beneficial for both patients and health care providers.

### Implementing a Remote Alert Alarm System

The initial costs of remote alert alarm system implementation were high since we had to equip each dialysis machine with a medical grade touch screen computer. Each computer cost $2,000 US, and we acquired 15 of them. However, newer dialysis machine models come equipped with on-board computers and WIFI for screen sharing with the dialysis machine, eliminating the need to purchase all-in-one medical grade computers. It took us only 3 months to develop software and to coordinate with Henry Ford Health System IT to integrate our development with the hospital phone system, and the remote alert alarm system is portable, since the dialysis machine interface with the computer is already functional. For hospitals that may want to create a similar system, interfacing with a hospital’s WIFI should be relatively simple, but phone system integration may be a challenge, albeit an achievable one. Thus, patient safety may be enhanced by leveraging existing technologies at nominal institutional expense.

The remote alert alarm system, by ideally reducing alarm resolution time, may ultimately liberate technicians and nursing staff for attending to other critical duties. The system can also be leveraged for increasing clinical communication. Adjunct to an alarm tote board, we created a web portal to display a running list of patients currently undergoing critical care hemodialysis. The list is refreshed every 5 minutes through remote alert alarm system monitoring of the dialysis machines that are actively treating patients, allowing the attending staff to easily determine the location of individuals undergoing critical care dialysis throughout the hospital.

Adding beneficial features to existing equipment by marrying technologies gives end users greater rewards and has the potential to increase patient safety. Melding current technology to create a new paradigm in dialysis machine alarm response by integrating WIFI with phone communication is a novel solution for enhancing patient safety. Unresolved dialysis alarms can lead to dire consequences for patients, and approaches to minimize risk are desired. Using SLED-RCA for continuous kidney replacement therapy combined with the remote alert alarm system alert approach has produced very few problems in the last 6 years, and our institution will continue to dynamically monitor and optimize these systems for delivering excellent care for patients who need dialysis.

### Limitations

Our quality improvement assessment of the remote alert alarm system dialysis machine alert approach has several limitations. First, the remote alert alarm system is a convenience feature that we implemented to allow technicians to track alarms and direct them to the location and type of alarm, and we did not plan a formal research study to assess generalizable results. We were not able to measure improvement compared to the previous system since that system had not been monitored previously, and data are routinely removed from the database for technical reasons. Therefore, our results regarding perceived improvements after system implementation are qualitative. Overall, this project was a technological enhancement of a communication system, and our findings are mainly anecdotal.

### Conclusion

Implementation of the remote alert alarm system as a remote alert system for monitoring dialysis machine alarms may increase technician efficiency and may be a way to reduce the time for responding to machine alarms. The remote alert alarm system may also save nursing time and prevent potentially dangerous situations from evolving, since it eliminates several tasks that nurses previously had to perform when responding to dialysis machine alarms. A remote real-time system for alerting hemodialysis technicians about machine issues may allow health care providers to be more responsive to alarm situations and expedite resolutions, reducing noise in the ICU and reducing patient risk while improving clinical care.
